# Is Obesity a Problem in New Cystic Fibrosis Treatments?

**DOI:** 10.3390/nu16183103

**Published:** 2024-09-14

**Authors:** Marta Solís-García, Marta María García-Clemente, Claudia Janeth Madrid-Carbajal, Adrián Peláez, Rosa Mar Gómez Punter, Jose María Eiros Bachiller, Rosa María Girón Moreno

**Affiliations:** 1 Servicio de Neumología, Hospital Universitario La Princesa, 28006 Madrid, Spain; marta.solis@salud.madrid.org (M.S.-G.); rosamar.gomez@salud.madrid.org (R.M.G.P.); josemaria.eiros@salud.madrid.org (J.M.E.B.); rosamaria.giron@salud.madrid.org (R.M.G.M.); 2 Instituto de Investigación Sanitaria del Principado de Asturias (ISPA), Universidad de Oviedo, 33011 Oviedo, Spain; 3 Servicio de Neumología, Hospital Universitario Central de Asturias (HUCA), 33011 Oviedo, Spain; claudiajaneth.madrid@sespa.es; 4 Facultad de Ciencias de la Salud-HM Hospitales, Universidad Camilo José Cela, 28014 Madrid, Spain; apelaez@fundacionhm.com

**Keywords:** cystic fibrosis, elexacaftor/tezacaftor/ivacaftor, CFTR modulator, nutrition, body mass index (BMI), lung function

## Abstract

Introduction: Malnutrition has always been a problem in CF (cystic fibrosis) patients; however, new treatments with CFTR (cystic fibrosis transmembrane conductance regulator protein) modulators have led to weight gain, with some patients at risk of overweight and obesity. Objective: Our study aimed to analyze the evolution of BMI (body mass index) after one year of treatment with triple therapy and the factors associated with weight gain in CF patients undergoing treatment with triple therapy with CFTR protein modulators (ETI) (elexacaftor/tezacaftor/ivacaftor). Methods: We conducted a prospective, observational, longitudinal, multicenter study in patients diagnosed with cystic fibrosis, aged 18 years or older, with at least one F508del allele and who underwent ETI therapy for at least one year, from 2020 to 2023. One hundred and eight patients from two cystic fibrosis units in Spain, Princess University Hospital of Madrid (74 patients) and Central University Hospital of Asturias (HUCA) (34 patients), were included. Demographic data, anthropometric data, lung function, and exacerbations were collected, comparing the data in the previous year to the start of therapy with the results after one year of treatment. Multivariant models were developed to account for repeated weight and BMI measurements, using a mixed effects model approach and accounting for possible modifying factors Results: One hundred and eight patients were included in the study, 58 men (53.7%) and 50 women (46.3%) with a mean age of 29.5 ± 9.4 years (18–59). Patient weight and BMI were recorded at baseline and at 3-month intervals during the study period. The weight increased from 59.6 kg to 62.6 kg and BMI increased from 21.9 kg/m^2^ to 23.0 kg/m^2^ after one year of treatment (*p* < 0.0001 for both). The proportion of underweight individuals decreased after one year of ETI therapy, from 9.3% to 1.9%, while the proportion of overweight or obese individuals increased from 8.3% to 22.9 % at the same time (*p* < 0.001). In relation to exacerbations, there is a significant increase in the number of patients who did not have any exacerbations after one year of treatment, which increased from 10.2% to 46.2% (*p* < 0.001), while the number of patients who had >4 exacerbations decreased significantly, from 40.7% to 1.9% (*p* < 0.001). FEV1% (forced expiratory volume) increased from 63.9 ± 20.9 to 76.8 ± 21.4 (*p* < 0.001) and the VR/TLC (residual volume/total lung capacity) value decreased from 45.1 ± 10.9 to 34.9 ± 6.2 (*p* < 0.001). The proportion with FEV1% > 80% increased from 23.1% before ETI therapy to 49.1% one year after ETI therapy. We performed multivariate mixed models to evaluate the evolution of BMI changes with time, accounting for repeated measures and for possible modifying factors. After the introduction of the triple therapy, patients included in the study had significant weight gain during the 12 months, and when including different covariates in the multivariate mixed model, we found that lower baseline BMI, lower baseline FEV1 and FVC (forced vital capacity), and higher VR/TLC value and higher number of exacerbations were associated with higher BMI changes over the study period. Conclusions: CF patients treated with triple therapy experience significant weight gain, increasing the proportion of overweight patients. CF patients who experienced greater weight gain were those with worse BMI at the start of treatment, as well as patients with worse lung function and a greater number of exacerbations in the year before starting ETI therapy.

## 1. Introduction

Cystic fibrosis is a life-shortening, genetic multi-system organ disease that affects 100.000 people worldwide [1,2] and whose clinical manifestations are due to mutations in the cystic fibrosis transmembrane conductance regulator (CFTR). The hepatic, pancreatic, and gastrointestinal alterations are noteworthy, although lung involvement marks the prognosis of the disease and determines survival [1,3]. Progressive structural lung damage, bronchiectasis, and chronic bronchial infection lead to a significant deterioration in lung function, with the FEV1% (forced expiratory volume) value being the most important single predictor of survival [3].

The nutritional situation is another key factor that impacts survival and is directly related to impaired pulmonary function and increased mortality [4,5,6,7]. Exocrine pancreatic insufficiency and high energy expenditure associated with the work of breathing and other factors, with greater severity when the disease is very advanced, contribute to these patients having lower body weight and body mass index (BMI) than age-matched controls. For this reason, one of the main objectives in managing these patients is to achieve a good nutritional situation, and guidelines from the Cystic Fibrosis Foundation recommend that adult women maintain BMI ≥ 22 kg/m^2^ and adult men BMI > 23 kg/m^2^ [8,9].

In recent years, there have been important changes in disease management with the initiation of treatment with CFTR protein modulators, achieving a significant improvement in lung function parameters. The nutritional situation has evolved in parallel, which has led to a significant increase in the patient’s survival. In this way, it is estimated according to the 2022 CF Foundation Annual Data Report, that people born with cystic fibrosis in 2021 will have a median life expectancy of 65.6 years and it is possible that in the future they will achieve estimated survival similar to the general population [4,10].

Triple therapy with the combination of elexacaftor/tezacaftor/ivacaftor (ETI) has led to a greater improvement both in terms of lung function and nutritional status in patients with at least one Phe508del allele [4,11,12]. In published randomized clinical trials, a weight gain of 1.13 points in BMI compared to 0.09 for the placebo group has been observed in 24-week observation studies [13]. Petersen et al. [6] found an increase of 1.47 kg/m^2^/yr (95% CI 1.08–1.87) in BMI after the start of ETI, and Carnovale et al. [14], found an increase from 20.9 kg/m^2^ to 23 kg/m^2^. Finally, Carrasco et al. [12] in a Spanish study of patients with advanced disease, found an increase in BMI from 20.5 to 22.3 kg/m^2^ after one year of ETI treatment.

In the current situation, we are faced with an increase in survival in these patients, but also and emergence of overweight/obesity as a potential new problem with the consequent cardiovascular risk, which has led to a new area of interest. Our study aimed to analyze the evolution of BMI after one year of treatment with ETI and the factors associated with weight gain in patients diagnosed with CF undergoing treatment with triple therapy with CFTR protein modulators (ETI).

## 2. Material and Methods

### 2.1. Study Design and Data Collection

We conducted a prospective, observational, longitudinal, multicenter study in patients diagnosed with cystic fibrosis, aged 18 years or older, with at least one F508del allele and who underwent treatment with ETI for at least one year, from 2020 to 2023. One hundred eight patients from two cystic fibrosis units in Spain, Princess University Hospital of Madrid (74 patients) and Central University Hospital of Asturias (HUCA) (34 patients), were included.

The study was approved by the Clinical Research Ethics Committee of both hospitals (CEIM Ref No 957/2020 and 067/2020) and informed consent was obtained from all included patients to participate in the study.

Data were prospectively collected at baseline and after 6 months and one year from ETI initiation.

### 2.2. Clinical and Demographic Variables

Patients were seen and evaluated in the specific CF clinic from clinical, functional, and nutritional perspectives at each visit. For each patient, demographic data (age and sex), genetic data, anthropometric data (weight, height and BMI), and pancreatic status were collected. In all cases, pulmonary function measurements were also collected (FVC—forced vital capacity, FVC%, FEV1, and FEV1% measured by spirometry and TLC%—total lung capacity, VR%—residual volume, and VR/TLC measured by plethysmography). Exacerbations were recorded one year before the ETI start and one year after ETI treatment. A sweat chloride test was performed for the 34 patients from HUCA (University Hospital from Asturias) before and 6–12 months after starting ETI.

Referring to comorbidities, pancreatic insufficiency was defined in patients treated with pancreatic enzymes, while CF-related diabetes (CFRD) was diagnosed according to an oral glucose tolerance test.

Exacerbation numbers were collected in the year previous to ETI start and after one year of ETI therapy.

The weight group designations were as follows [15]:

- BMI < 18.5 kg/m^2^ (underweight).
- BMI 18.5–24.9 kg/m^2^ (target weight).
- BMI 25–29.9 kg/m^2^ (overweight).
- BMI > 30 kg/m^2^ (obese).

### 2.3. Statistical Study

Statistical analysis was performed using SPSS 23.0 (IBM Corp, Armonk, NY, USA) and R 4.1.0 (R Foundation for Statistical Computing, Vienna, Austria). Descriptive analysis was performed using means and standard deviations or medians and interquartile ranges for quantitative variables and proportions and percentages for qualitative variables. The normality in the distribution of the variables has been determined by the Kolmogórov–Smirnov test. Bivariant analysis was performed using linear regression and Pearson/Spearman coefficients, logistic regression, and ANOVA tests for repeated measures. Multivariant models were developed to account for repeated weight and BMI measurements, using a mixed effects model approach and accounting for possible modifying factors that included time, age at the start of the study, baseline pulmonary function tests, pancreatic insufficiency, type of mutation, and number of exacerbations in the previous year. Statistical significance was set at *p* < 0.05.

## 3. Results

One hundred eight patients were included in the study, 58 men (53.7%) and 50 women (46.3%) with a mean age of 29.5 ± 9.4 years (18–59). All patients included in the study received at least one year of ETI treatment, and different parameters were evaluated after one year of ETI therapy. Table 1 details the baseline characteristics of the study sample.

### 3.1. BMI

Patient weight and BMI were recorded at baseline and 3-month intervals during the study period. Table 2 summarizes the changes in patient weight and BMI (from 59.6 kg to 62.6 kg and from 21.9 kg/m^2^ to 23.0 kg/m^2^, respectively) (*p* < 0.0001 for both).

**Figure 1:**
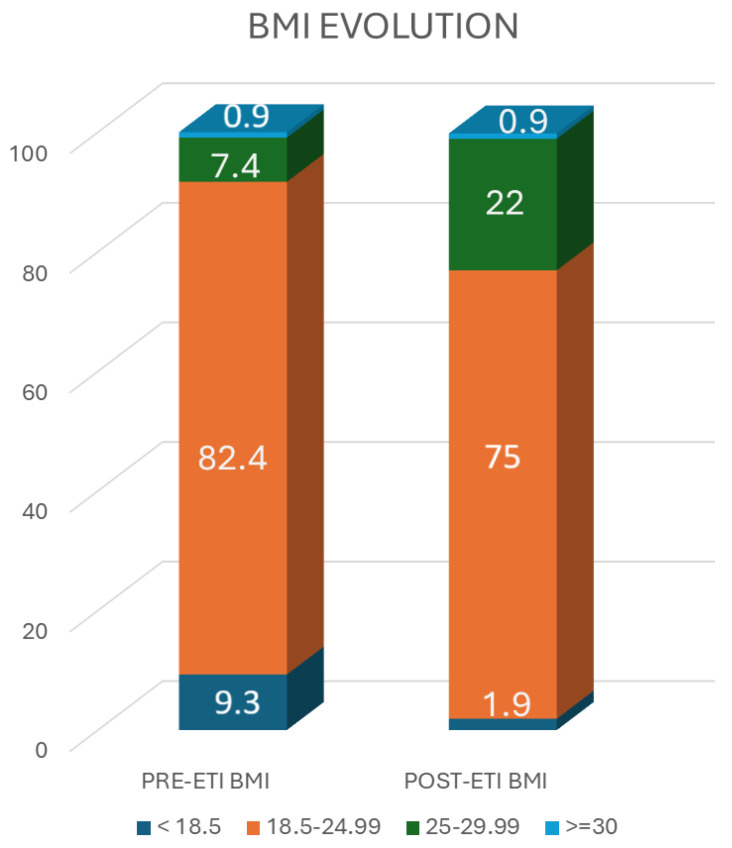
BMI evolution concerning BMI designations of the group (kg/m^2^).

When we analyzed the BMI evolution concerning the weight group designation, we observed that before the initiation of ETI treatment, 9.3% were underweight (BMI < 18.5 kg/m^2^), 82.4% of target weight (BMI: 18.5–24.9 kg/m^2^), 7.4% overweight (BMI: 25–29.9 kg/m^2^), and 0.9% obese (BMI ≥ 30 kg/m^2^). After one year of ETI treatment 1.9% were underweight (BMI < 18.5 kg/m^2^), 75% were target weight (BMI: 18.5–24.9 kg/m^2^), 22% were overweight (BMI: 25–29.9 kg/m^2^), and 0.9% obese (BMI ≥ 30 kg/m^2^). The proportion of underweight individuals decreased during one year of ETI therapy, from 9.3% to 1.9%, while the proportion of overweight or obese individuals increased from 8.3% to 22.9% at the same time (*p* < 0.001) (Figure 1).

### 3.2. Sweat Chloride Test

**Figure 2:**
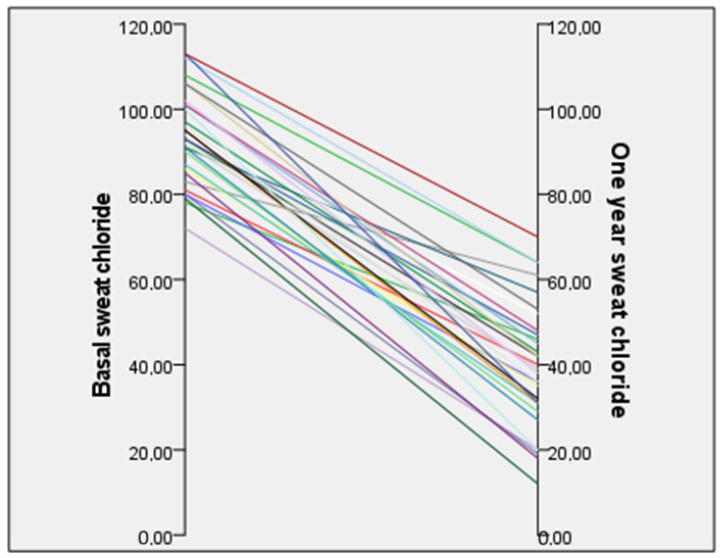
Decrease in sweat chloride test values in the patients analyzed (derived from HUCA hospital; *n* = 34 patients). Paired *t*-test (*p* < 0.001).

Sweat chloride was performed on the 34 patients from one of the center participants in the study (HUCA). Overall, mean sweat chloride concentration decreased from 93.5 ± 10.8 mmol/L at the start of ETI treatment to 39.8 ± 14.5 mmol/L within one year of treatment (change of −53.6 mmol/L; 95% confidence interval −49, −58.3) (*p* < 0.001); reductions were similar after 6 months of treatment; mean sweat chloride concentration decreased from 93.5 ± 10.8 at the start of ETI treatment to 36.1 ± 16.4 (change of −57.4 mmol/L; 95% confidence interval −51.6, −63.2) (*p* < 0.001). In Figure 2, the decreases in the sweat test values of the 34 patients analyzed are observed.

### 3.3. Exacerbations

**Figure 3:**
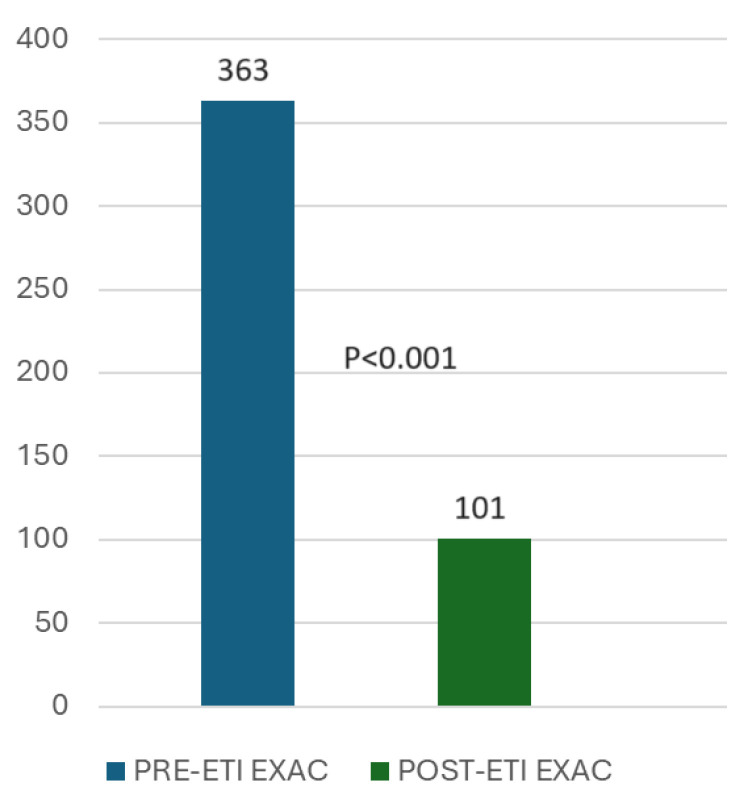
Decreased exacerbation number after one year of ETI treatment. Paired *t*-test (*p* < 0.001).

There were 363 exacerbations in the year before starting ETI treatment and 101 exacerbations in the year after ETI therapy initiation (−72.2%; *p* < 0.001) (Figure 3).

**Figure 4:**
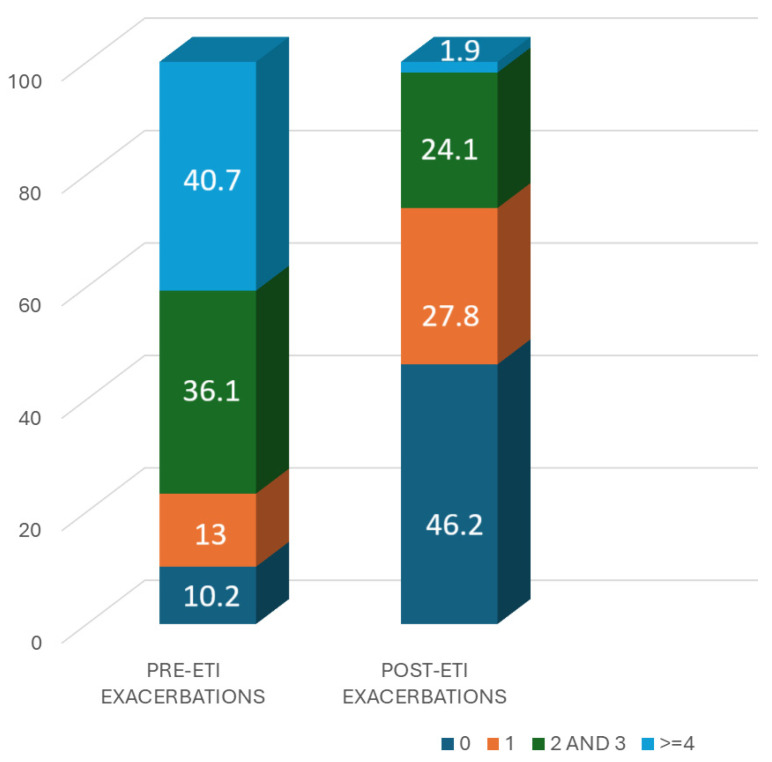
Exacerbation number per subject (McNemar *p* < 0.001).

Figure 4 shows the number of patients with 0, 1, 2–3, and ≥4 exacerbations in the previous year and the year after the start of ETI treatment. There is a significant increase in the number of patients who did not have any exacerbations, which increased from 10.2% to 46.2% (*p* < 0.001), while the number of patients who had >4 exacerbations decreased significantly, from 40.7% to 1.9% (*p* < 0.001).

The number of total exacerbations, exacerbations treated with oral antibiotics or intravenous antibiotics, and the number of admissions decreased significantly after one year of ETI treatment (Table 3).

### 3.4. Lung Function

**Figure 5:**
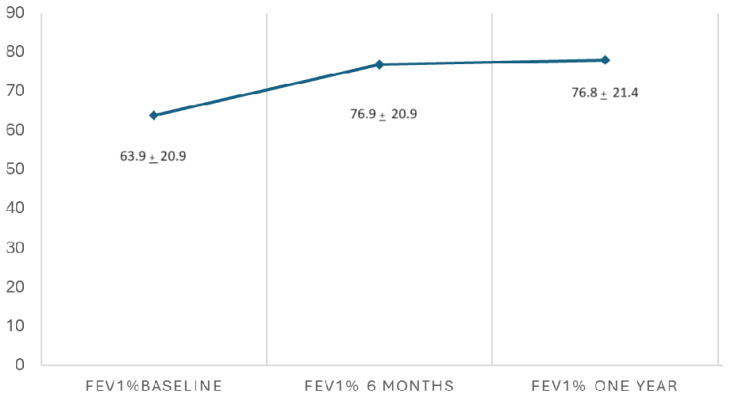
FEV1% evolution after one year of ETI therapy. Paired *t*-test (*p* < 0.001).

**Figure 6:**
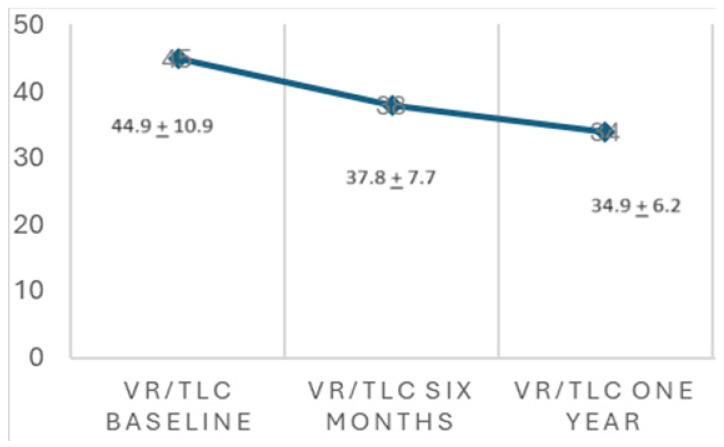
VR/TLC evolution after one year of ETI therapy. Paired *t*-test (*p* < 0.001).

A significant improvement in FEV1% and FVC% values was observed after one year of ETI therapy. FEV1% increased from 63.9 ± 20.9 to 76.8 ± 21.4 (*p* < 0.001) (Figure 5). The FVC% value increased from 79.9 ± 16.9 to 90.5 ± 15.1 (*p* < 0.001). The VR/TLC value decreased from 45.1 ± 10.9 to 34.9 ± 6.2 (*p* < 0.001) (Figure 6). The proportion of CF patients with FEV1% in lower categories decreased during ETI therapy, while the proportion in higher function categories increased. The proportion with FEV1% > 80% increased from 23.1% before ETI therapy to 49.1% one year after ETI therapy.

### 3.5. BMI Change during the Study Period

**Figure 7:**
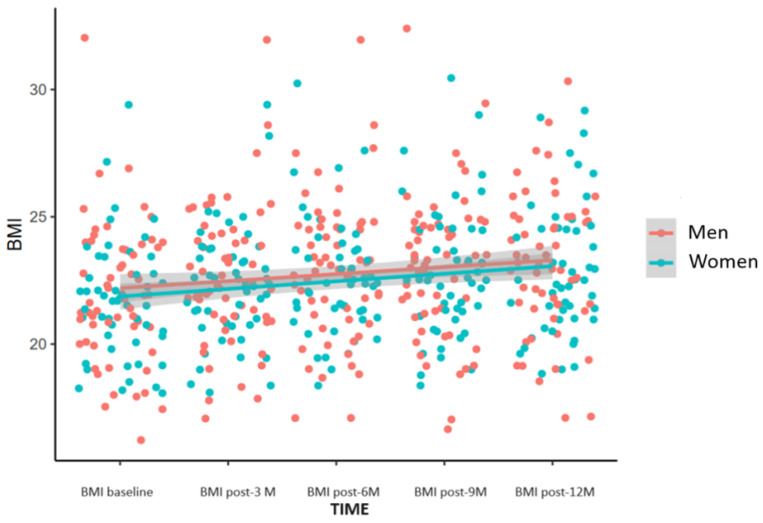
BMI change during the period of study in men and women. Paired *t*-test (*p* < 0.001).

Figure 7 shows a progressive increase in BMI after the ETI therapy started in both men and women.

Univariant correlations were made through linear and logistic regression between the different baseline characteristics and the change in BMI during the study period (Table 4).

In univariate analysis, the improvement in pulmonary function was correlated with changes in BMI (Table 5).

We performed multivariate mixed models to evaluate the evolution of BMI changes with time, accounting for repeated measures and possible modifying factors. After the introduction of the triple therapy with ETI, patients included in the study had significant weight gain during the 12 months, and when including different covariates in the multivariate mixed model, we found that lower baseline BMI, lower baseline FEV1 and FVC, and higher VR/TLC value and higher number of exacerbations were associated with higher BMI changes over the study period (Table 6).

**Figure 8:**
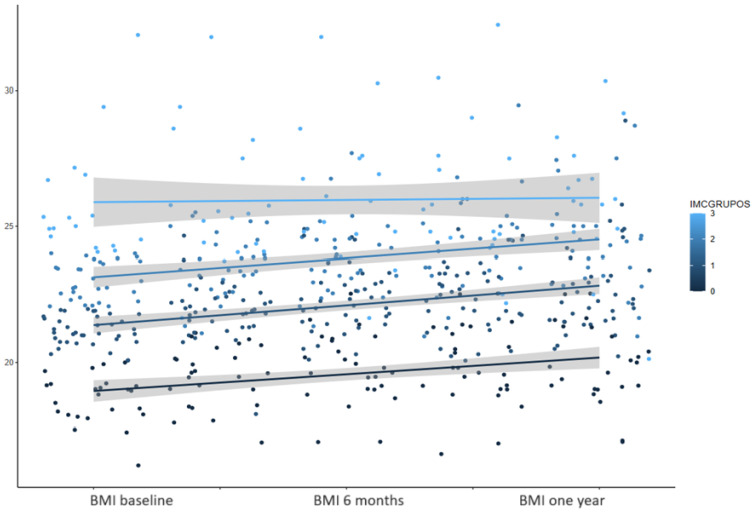
Baseline BMI influences the evolution of BMI throughout the study: the 4 groups (<20, 20–22, 22–24, and >24).

BMI increase over the one year after the start of ETI therapy was significantly higher in those patients who had lower BMI values at the ETI therapy start (Figure 8). This figure shows that there is more improvement in the groups with BMI below 24 kg/m^2^.

**Figure 9:**
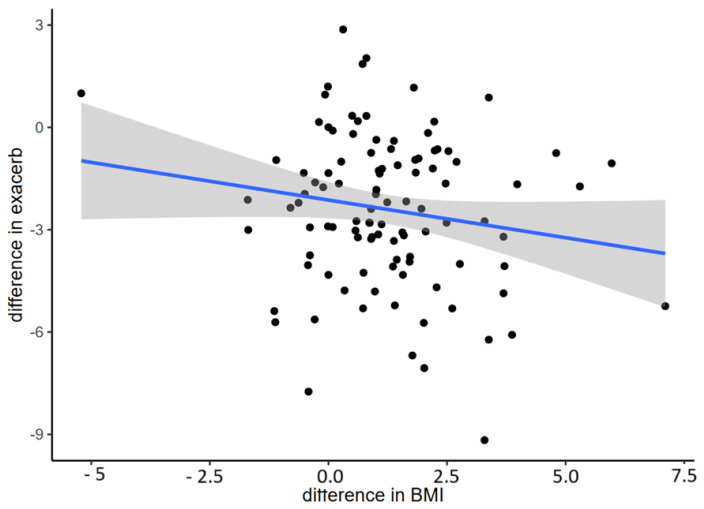
Relation between changes in BMI and difference in exacerbations (*p* = 0.02). The blue line expresses the relationship between the difference in exacerbations and the change in BMI. Shadows express the concentration or dispersion of the data.

Weight gain was associated with a decrease in exacerbations and admissions, but results were statistically significant only in exacerbations (Figure 9).

## 4. Discussion

Nutritional care has been a key element in the management of patients with cystic fibrosis, with malnutrition being one of the factors that has had the greatest impact on respiratory functional deterioration. However, the start of treatment with CFTR protein modulators has led to a significant increase in BMI values, and an increase in the prevalence of overweight and obesity [8,16] has even been observed in this population with a classical tendency towards malnutrition. In our study, we have observed an increase in BMI of 1.1 kg/m^2^ after one year of ETI therapy with an increase in overweight/obesity from 8.3% in the pre-ETI therapy period to 22.9% after one year of ETI treatment (*p* < 0.001). Weight gain was significantly greater in patients with lower BMI at the beginning of treatment and in those with worse lung function and a greater number of respiratory exacerbations. Detecting the factors associated with this weight gain is essential to prevent overweight and obesity, which can increase cardiovascular risk, diabetes, cancer, and psychosocial alterations derived from obesity.

This weight gain has already been described in other studies as one of the parameters to highlight in the ETI treatment [4,11,12,13]; however, results are very heterogeneous and the mechanisms by which it occurs are unknown. Some authors postulate that the decrease in energy expenditure at rest, increased calorie consumption, and improved intestinal absorption play an important role in the weight gain in adult patients with CF on treatment with CFTR protein modulators [8,17]. On the other hand, patients have a higher fat intake taking into account that the recommendation for ETI administration is to take it with a high-fat meal twice a day for better absorption of the modulating treatment, although the quantity of fat that should be consumed for optimal drug efficacy is unknown. Other factors that may be related to weight gain are improvement in the exocrine pancreatic function experienced by some patients [18] and the lower energy expenditure related to respiratory work, more evident in those patients with advanced disease [19,20].

In our study, the baseline BMI value prior to the start of ETI therapy was the factor that had the greatest impact on weight gain. Other studies, such as that of Gramegna et al. [19], show similar results, with the baseline BMI being the main determinant of heterogeneity in the response to treatment in this study, with a greater increase in the population of patients with CF with lower BMI values before the start of ETI therapy. In our study, post-treatment changes in BMI were significantly higher in both the underweight and target-weight groups, but there was no evidence of weight gain among patients with overweight [19].

Another factor to consider in relation to weight gain, already observed in other studies, has been lung function, which has always been greatly influenced by the nutritional situation. For this reason, nutritional care has been a key factor in improving the survival of these patients [8]. However, the presence of overweight and obesity has not been related to an improvement in lung function. It is important to highlight that overweight has been associated with improvement in lung function and the number of exacerbations in CF patients, but these benefits have not been observed when BMI rises above a threshold of 28–29 kg/m^2^ [21]. In the Canadian registry, an increase in lung function has been observed with weight gain; however, the magnitude of this increase was significantly lower in obese patients than in those whose weight was within the reference range [22]. In this sense, various studies have shown that the presence of overweight and obesity are not protective factors and do not confer benefits to lung function [8,22,23]. In this sense, there is evidence that in children with CF with pancreatic sufficiency, a BMI > 85th percentile has a detrimental effect on pulmonary function [24]. Our data coincide with those of Stewart et al. [4], observing in our study greater weight gain in those patients with worse lung function and those with greater air trapping at the start of ETI therapy. The improvement in air trapping can lead to greater weight gain, probably by achieving better ventilatory mechanics and therefore less respiratory work with lower caloric consumption.

In our study, the greater number of exacerbations before the start of ETI therapy was related to greater weight gain after one year of treatment in the models found in linear mixed effects modeling. In the study by Carnovale et al. [14], a gain of 2.08 kg/m^2^ was observed in patients with more advanced disease, who are those with the highest exacerbation number. The same data are collected in the Spanish study published by Carrasco et al. in which an increase of 2.8 kg/m^2^ was observed in patients with advanced disease with a decrease in the total exacerbation number from 3.9 to 0.9 (*p* < 0.001) [12]. In the study by Harindhanavudhi et al. [21], overweight patients had fewer exacerbations and the highest frequency of exacerbations was experienced by underweight patients, observing an inverse relationship between BMI and frequency of pulmonary exacerbations. Similar results were obtained by Stewart et al. [4], showing greater weight gain in those patients with a greater number of exacerbations in the previous year.

All these factors must be taken into account when controlling the development of overweight and obesity to avoid the development of cardiovascular problems and an increase in the incidence of diabetes, cancer, and psychosocial problems derived from obesity. In patients with CF, there are few studies about how obesity can affect the risk of developing cardiovascular diseases, diabetes, or cancer, given that the life expectancy of these patients to date has not made it possible to evaluate these studies. However, in the current situation, with a significant increase in life expectancy, it will be a priority to analyze how the development of overweight and obesity can influence the development of these comorbidities [8,23,25,26]. In the study carried out by Harindhanavudhi et al. [21], the prevalence of hypertension was 31% in overweight and 25% in obese patients compared to 17% in target-weight CF adults (*p* = 0.01). Overweight and obese patients had statistically higher total cholesterol, LDL cholesterol, and triglyceride levels compared to underweight and target-weight patients after adjustment for sex and age.

Finally, despite the great improvement experienced by CF patients with treatment with CFTR protein modulators, lung transplantation continues to be a treatment option for end-stage disease. Evidence suggests that both overweight and malnutrition are associated with higher mortality rates following a lung transplant, so BMI ≥ 35 kg/m^2^ is considered an absolute contraindication to lung transplant, and BMI 30–34.9 kg/m^2^ a relative contraindication [8].

## 5. Conclusions

In conclusion, CF patients treated with ETI therapy experience significant weight gain, increasing the proportion of overweight and obese patients. CF patients who experienced greater weight gain were those with worse BMI at the start of treatment, as well as patients with worse lung function and a greater number of exacerbations in the year before starting ETI therapy. Additionally, the presence of overweight and obesity is not associated with greater improvements in lung function, and although over the last decades, much emphasis has been placed on promoting a good nutritional situation to optimize lung functional evolution, the risk of overweight/obesity is elevated in CF patients with ETI therapy and therefore must be monitored long term. It is important to insist on good dietary habits and the need for physical exercise in these CF patients with risk factors for greater weight gain [27].

It will be necessary to promote studies based on registries in order to determine the longitudinal association between the BMI increase and pulmonary functional evolution in the CF population. Identifying the factors associated with this weight gain may be important with a view to preventing the consequences derived from overweight and obesity, given the risk of the development of many chronic and potentially life-threatening health conditions such as cardiovascular diseases, cancer, diabetes, and psychosocial disorders. On the other hand, it will be of great importance to understand the underlying mechanisms and factors associated with this weight gain above what is considered reasonable to establish individualized dietary recommendations in these CF patients with a greater tendency to overweight and obesity.

As limitations of the study, we can highlight that it has only been carried out in two CF centers in the country and patients were only followed for one year, so there is a possibility that CFTR protein modulators may behave differently over the years. On the other hand, only the BMI has been taken into account, without having assessed the body composition data, which could be more useful than the BMI, although it is easier to generalize the results as it is a parameter that is easy to measure and is collected in all CF patients.

The strengths that can be highlighted are that it is a prospective study in which all the variables included in the study have been collected in detail. On the other hand, the study includes a moderately large sample size with sizable cohorts of both F508del homozygotes and heterozygotes.

## Figures and Tables

**Table 1:** Baseline characteristics of the study sample.

Characteristics	
Age (mean ± SD)	29.5 ± 9.4 (18–59)
Sex, female	50/108 (46.3%)
BMI baseline	21.9 ± 2.5
Type of mutation	
F508del homozygous	49/108 (45.4%)
F508del heterozygous	59/108 (54.6%)
Exocrine pancreatic insufficiency	91/108 (84.3%)
Endocrine pancreatic insufficiency	45/108 (41.7%)
Previous treatment with a modulator	46/108 (42.6%)
Total number of exacerbations in the previous year	3.4 ± 2.3 (0–10)
Treated with oral antibiotics	2.7 ± 1.9 (0–9)
Treated with intravenous antibiotics	0.6 ± 1.1 (0–8)
Total number of admissions in the previous year	0.3 ± 0.6 (0–4)
FVC% baseline	80.1% (SD 16.9)
FEV1% baseline	64.3% (SD 20.9)
VR% baseline	181 ± 67 (94–352)
VR/TLC baseline	44.9 ± 10.9 (27.4–61.6)
Sweat chloride test	93.5 ± 10.8 (72–113)

Qualitative variables are presented as frequencies and percentages. Quantitative variables are presented as means and standard deviations (SDs). FVC: forced vital capacity; FEV1: forced expiratory volume first second; VR: residual volume; VR/TLC: residual volume/total lung capacity.

**Table 2:** Differences in weight and BMI during the study period.

	Weight	BMI
Baseline	59.6 kg (9.8)	21.9 (2.5)
3 months	61.7 kg (10.0)	22.5 (2.5)
6 months	62.4 kg (10.2)	22.8 (2.6)
9 months	62.9 kg (10.4)	22.9 (2.7)
12 months	62.6 kg (10.2)	23.0 (2.7)
*p*-values	<0.0001	<0.0001

Values are presented as means and standard deviations (SDs), and *p*-values reflect statistical analysis with a univariant ANOVA post hoc test for repeated measurements.

**Table 3:** Decreased in the exacerbations number.

	Exacerbations	Oral Antibiotics	IV Antibiotics	Admissions
Previous year	3.36 ± 2.33 (0–10)	2.73 ± 1.90 (0–9)	0.58 ± 1.15 (0–8)	0.26 ± 0.59 (0–4)
One year post-ETI	0.94 ± 1.16 (0–7)	0.83 ± 1.09 (0–7)	0.09 ± 0.44 (0–3)	0.06 ± 0.33 (0–3)
*p*-values	<0.001	<0.001	<0.001	<0.001

IV: intravenous. ETI: elexacaftor/tezacaftor/ivacaftor. Paired *t*-test (<0.001).

**Table 4:** Correlation coefficients for BMI increase.

Characteristics	Correlation Coefficient	*p*-Value
Sex	−0.01 (−0.25; 0.28)	0.98
Type of mutation	0.19 (−0.19; 0.64)	0.37
Exocrine pancreatic insufficiency	−0.05 (−0.41; 0.33)	0.75
Endocrine pancreatic insufficiency	0.08 (−0.17; 0.37)	0.91
Previous treatment with a modulator	−0.04 (−0.28; 0.21)	0.76
Age at the start of the triple treatment	−0.03 (−0.16; 0.11)	0.79
Total number of exacerbations in the previous year	0.16 (0.06; 0.26)	0.01
Total number of admissions in the previous year	0.09 (−0.12; 0.28)	0.37
FVC %, baseline	−0.10 (−0.32; 0.11)	0.29
FEV 1%, baseline	−0.20 (−0.37; −0.02)	0.04
Baseline BMI	−0.25 (−0.41; −0.05)	0.01
Baseline VRTLC	0.33 (0.11; 0.68)	0.03

Correlation coefficients correspond to linear regression for quantitative variables and logistic regression for binomial variables. Bold values represent statistical significance.

**Table 5:** Improvement in pulmonary function was correlated with changes in BMI.

Pulmonary Function Test, Changes	Correlation Coefficient with Changes in BMI	*p*-Value
Changes in FEV1, %	0.22 (0.03; 0.41)	0.02
Changes in FVC, %	0.27 (0.07; 0.44)	0.006
Changes in VR/TLC	−0.45 (−0.75; −0.04)	0.03
Changes in VR, %	−0.39 (−0.77; 0.08)	0.07
Changes in TLC, %	−0.22 (−0.63; 0.22)	0.31

**Table 6:** Multivariable model of BMI change in one year.

Multivariate Mixed Model	*p*-Value
Time	<0.001
BMI at baseline	<0.001
Baseline FEV1, %	0.001
Baseline FVC, %	0.03
Baseline VR/TLC	0.02
Exacerbations	0.02

## Data Availability

Data are unavailable due to privacy or ethical restrictions, but they are available if someone wants to have them for verification or meta-analysis.
